# Surgery

**Published:** 1898-02

**Authors:** John Parmenter

**Affiliations:** Buffalo, N. Y., Professor of anatomy and adjunct professor of clinical surgery, Buffalo University Medical College


### Progress in Medical Science.

### SURGERY.

Conducted by JOHN PARMENTER, M. D., Buffalo, N. Y.,
Professor of anatomy and adjunct professor of clinical surgery, Buffalo University Medical College.

### FIVE CASES OF GENERAL SUPPURATIVE PERITONITIS TREATED BY A NEW METHOD.

FINNEY (*Johns Hopkins Hospital Bulletin*) bases his operation upon the known power of the healthy peritoneum to take up and dispose of infectious material. He regards this property as possessed by the infected peritoneum as well, and that operation in septic peritonitis often fails because the peritoneum is not sufficiently cleansed at the time of operation. The steps of the operation described by him are briefly as follows : A sufficiently long incision to permit easy access to the peritoneal cavity is made, the coils of the small intestine are removed from the peritoneal cavity, beginning with the worst coils first ; these are packed in warm gauze or towels and then thoroughly and systematically he wipes out the peritoneal cavity with large pledgets of gauze wrung out of hot salt solution, paying particular attention to the pelvic portion of the cavity. Next the small intestines are systematically examined, loop by loop, and cleansed by wiping with gauze compresses wrung out with hot salt solution as before. All adherent flakes of partly organised lymph must be entirely removed, considerable force being sometimes necessary to effect this. Upon the thoroughness of this cleansing, both of the intestines and peritoneal cavity, the success of the operation depends. It is advantageous to wipe the coils under a constant irrigation of warm salt solution. All foreign material having been removed, the intestines being thoroughly clean to the naked eye, the bowels are returned, the most damaged portion of the intestine being returned last, this giving it a superficial position. The abdominal wound is then tightly closed, room enough being left for a gauze drawn between two sutures. Distension or pain in the abdomen after the operation is treated by a thorough application of the Paquelin cautery ; calomel in divided doses, followed by a turpentine enema, are used for catharsis. An essential for success is that the operation should be performed within a few hours after perforation. Five successful cases are detailed to support the claims made for this method.

---

### DIFFERENTIAL DIAGNOSIS BETWEEN APPENDICITIS AND INTESTINAL OBSTRUCTION FROM GALL-STONES.

Th. Kölliker (*Centralblatt für Chirurgie*, October 28, 1897,) relates a case of obstruction from gall-stone presenting diagnostic difficulty and much resembling a case of appendicitis described by Sonnenberg in his work upon perityphlitis. The history as given is briefly as follows :

Woman, 58 years of age, without previous history of gall-stone colic, but having had a few months previously an attack of appendicitis, was suddenly taken ill with constipation three days previous to his first observation, followed in twenty-four hours by fecal vomiting ; a tumor could be felt in the ileo-cecal region. Various diagnoses were made by observers, one being compression of the bowel from an old perityphlitic exudate, another being obstruction due to disease of the uterine appendages of the right side. There were no symptoms of the trouble in the biliary passages. The author concluded that the case was one of compression of the bowel by a perityphlitic exudate. The peritoneal cavity was opened in the middle line. The right half of the cavity was filled with distended bowels and from the left distended coils of small intestine pressed forward. Presently a hard body, filling the lumen of the bowel, was discovered. The bowel was opened on its convex surface and a gall-stone, four centimeters by three centimeters, was extracted. The mucous membrane of the intestine was closely attached to the stone and ulcerated in the superficial surface. The wound was closed and recovery ensued, the first stool occurring twenty-four hours after operation.

For comparison, Sonnenberg's case was narrated and exhibited the following symptoms: The case was that of a fifty-year-old patient who had had several attacks of perityphlitis, and in recent times a resistance in the ileo-cecal region, with acute symptoms, and obstruction appeared. Fever was absent. After opening of the peritoneal cavity there was revealed a chronically inflamed and moderately adherent appendix, which seemed scarcely sufficient cause for new developments, especially as the previous resistance in this region had disappeared. After a search a goodly-sized gall-stone was discovered lying in one of the coils of the intestine. The patient died in collapse with reappearance of the obstruction before death. At the autopsy a second stone was found wedged in the duodenum, another one in an ulcerated point of communication between the gall-bladder and the duodenum, and a fourth in the gall-bladder. The coils from which the gall-stone had been removed were in part gangrenous.

The writer concluded that some cases are most difficult to diagnosticate before opening the abdomen, but that the greater movability of the tumor and the acute onset without fever are suggestive of gall-stone obstruction.

---

### THE USE OF THE PHONENDOSCOPE.

Van Arsdale, (*New York Polyclinic*, August 15, 1897,) after describing the instrument, proceeds to enumerate the various uses to which it may be put. Among these are the mapping out of various organs, the detection of fractures and dislocations, the diagnosis of tumors, and the like. An illustrative example of the method of using this instrument in detecting fractures of the ulna is given. The pin of the instrument is placed over the head of the ulna at the lateral aspect of the wrist, and a scratching or rubbing with the finger is made over the olecranon, the sound being readily transmitted unimpaired to the ears when the ulna is intact; if, however, a fracture has taken place in the shaft of the ulna the sound will be heard very indistinctly, and if the ends of the fragments be not in contact no transmission of sounds whatever will take place. Slight variations in transmission are readily appreciable on comparing the conditions in the sound arm with those in the injured one. In another instance the diagnosis of muscles lying between the fractured ends of the clavicle and preventing union was made sometime later with verification by operation.

Effusions in the knee-joint may be detected by placing the pin either as near as possible to the capsule of the joint or on the ligamentum patellæ, the vibrations in the fluid set in motion by friction of the finger being plainly perceptible. Tumors with density interfering with that of the neighboring tissues may be outlined and thus supplement palpation or be of value where palpation is impracticable. Tension having been properly estimated by palpation one can differentiate between the various sounds of transmission in solid matter, in fluids and in air. It is to be remembered that the greater density of the examined organ the greater the force required in stroking the finger with the skin to elicit transmission. The pitch also becomes deeper with the density. The author finds its most practicable value in the diagnosis of abdominal tumors, and relates instances where by its use he has diagnosticated tumors connected with the liver and with the ascending and transverse colon at the flexure. He also believes it perfectly practicable to differentiate intra- from retro-peritoneal tumors.

---

Joseph Price (*International Journal of Surgery*, October, 1897,) gives some forcible and undeniable conclusions upon the subject of post-operative complications and reoperations. The writer believes that there are too many useless and harmful operations being done at the present time, that reoperations are occasioned by one or two circumstances. In the first place, where complete operation might imperil the life of the patient ; in the other, reoperation is occasioned because of the ignorance or cowardice of the operator, and that this latter is by far the more frequent cause. He believes that recovery from operation should mean cure in the vast majority of cases, but that it does not is well known. Before operation there should be reasonable certainty as to the existing trouble in order that future action may be decided. In many cases repeated operation was unjustifiable and caused sequelæ which make the second operation much more difficult and dangerous. All exploratory operations he regards as implying ignorance and doubt, but when properly done there may be no deleterious after-results. Among the procedures giving a large per cent. of operations the writer mentions dilatation and curettement, vaginal puncture, vaginal hysterectomy, appendicitis, complicated by obstruction or adhesions following primary operation, neglect to free adhesions, e. g., from the bladder or ileum, in all cases where they exist after the primary operation, to neglect to remove the remaining and irritating materials; to trim away ragged and fringy adhesions, to clear away débris and clot discharges, to establish a sufficient drainage, and the like. The paper is well worthy the perusal of surgeons both young and old, and being as it is, unquestionably, based upon a large experience, its wholesome truths should become well known to surgeons.

